# Bias Adjustment Techniques Are Underutilized in HIV Sexual Risk Estimation: A Systematic Review

**DOI:** 10.3390/ijerph15081696

**Published:** 2018-08-09

**Authors:** Nguyen K. Tran, Neal D. Goldstein, Seth L. Welles

**Affiliations:** Department of Epidemiology and Biostatistics, Dornsife School of Public Health, Drexel University, Nesbitt Hall, 3215 Market St., Philadelphia, PA 19104, USA; nt448@drexel.edu (N.K.T.); ng338@drexel.edu (N.D.G.)

**Keywords:** HIV/AIDS, men who have sex with men, misclassification, exposure assessment

## Abstract

*Background:* Valid measurement of determinants of HIV infection among men who have sex with men (MSM) is critical for intervention planning and resource allocation. However, sexual minority research concerning HIV risk often relies on proxy exposures of sexual behaviors such as sexual orientation and partner gender. Inferring high risk sexual behaviors (i.e., condomless anal intercourse) from these proxies inaccurately captures HIV risk, but few studies have attempted to correct for this bias. *Methods:* We performed a systematic review of methodological practices for estimating risk of HIV infection among MSM. *Results:* We identified 32 studies in which high risk sexual behavior was assessed: 82% (*n* = 26) measured and used sexual risk behaviors (e.g., condomless anal intercourse or sexual positioning) to assess risk of HIV infection; 9% (*n* = 3) used proxy measures; and 9% (*n* = 3) used both behavior and proxy variables. Various treatments of misclassification reported by investigators included the following: 82% (*n* = 26) discussed misclassification of sexual behavior as a potential limitation; however, among these studies, no attempts were made to correct misclassification; 12% (*n* = 4) did not report exposure misclassification, and 6% (*n* = 2) explicitly considered this information bias and conducted a Bayesian approach to correct for misclassification. *Conclusions:* Our systematic review indicates that a majority of studies engaging in collecting primary data have taken additional steps to acquire detailed information regarding sexual risk behaviors. However, reliance on population-based surveys may still lead to potentially biased estimates. Thus, bias analytic techniques are potential tools to control for any suspected biases.

## 1. Introduction

Human sexuality is a complex construct, which involves considering three key aspects: sexual orientation (how individuals describe or identify their sexuality), sexual attraction (to whom an individual is sexually attracted regardless of biological sex or gender identity), and sexual behaviors (with whom an individual says they have sexual experiences) [1,2]. When evaluating HIV risk among gay and bisexual men, it is the actual sexual behavior (i.e., condomless anal sex) that transmits the pathogen and not how the individual identifies his or her sexuality [3]. 

Unfortunately, many generalizable surveys that attempt to capture population health at a broad level, such as the National Health and Nutrition Examination Survey (NHANES), collect data on sexual behaviors in a limited and problematic manner. Rather, they rely upon proxy measures of behavior, such as sexual orientation. The use of proxies may be intentional since these surveys need to balance breadth versus depth. Nevertheless, the data captured are not ideal for examining HIV transmission, since some men who identify as gay or bisexual may engage in low risk same-sex behaviors (e.g., oral sex). Further complicating the issue, surveys that collect socially sensitive data on potentially stigmatizing factors such as same-sex orientation and behavior are prone to underreporting [4,5], potentially compromising the important public health charge of accurately measuring HIV risk in high risk communities. 

One possible scenario is that people identify as heterosexual or straight, but also engage in sex with partners of the same gender [6]. In epidemiology, this phenomenon is known as misclassification, which threatens internal validity of study findings. It is important to note that this is not a misclassification of their orientation (being gay/homosexual or bisexual), but inferring sexual behaviors from self-reported sexual orientation can be problematic when assessing HIV risk. Any error introduced in exposure assessment is carried forward into the analytic modeling of the outcome and possibly rendering the predictions unreliable. 

### Consequences of Misclassifying Sexual Behavior in HIV Risk Estimation

Men that identify as gay, bisexual, or have sex with men (MSM) are overrepresented among new HIV cases in the United States [3]. As described, MSM behavior may be denoted by self-reported sexual orientation, same-sex attraction, sexual relationships with other men (same-sex partner), or a combination of these identifying factors. MSM from communities where same-sex behaviors and identities are stigmatized tend to conceal such information, resulting in misclassification of their potential risk for HIV transmission [7,8]. Further, this misclassification may be differential, in that knowledge of one’s HIV serostatus may affect how they report sexuality. Prior work has shown that individuals with and without a history of sexually transmitted infections have different probabilities of reporting gay identity or same-sex behaviors [7,8]. Accordingly, this differential misclassification may bias HIV risk estimates among gay and bisexual men. Research that relies on partner gender for assessing risk of transmitting or acquiring HIV is likely to be less biased than research that relies on reported sexual identity [9]. However, even partner gender may not capture risk entirely accurately; for example, an individual may have male sex partners but engage entirely in oral sex, conferring a lower risk for infection. 

Among transgender individuals, assessing this high-risk behavior through proxy variables is further complicated by the range of genital configurations; transgender individuals may or may not have same-sex attraction or identity regardless of their genitalia. Thus, rather than rely on sexual and/or gender identity, or partner gender, it is crucial to identify individuals who engage in receptive anal intercourse, which confers the greatest risk for infection. 

Without optimal data, researchers must rely upon bias correction methodology [10], but it is currently unclear how often these techniques are used in HIV and other sexually transmitted infections research to obtain less biased estimates of risk in sexual minority groups. In addition, Pathela et al. [6] and Igartua et al. [11] have heeded the need to improve exposure assessment of high risk sexual behaviors. This sentiment is relatively prevalent as several studies (approximately a total of 200 cited articles) have referenced these articles. Therefore, in this paper, we sought to examine how often assessment of risk of HIV transmission due to condomless anal intercourse is evaluated via commonly reported proxy variables (such as self-reported sexual orientation or gender of partners), further highlighting the need to adopt recent methodology to adjust for this misclassification. 

## 2. Methods 

We conducted a systematic review of the literature, targeting peer-reviewed publications that have reported methods for estimating risk of HIV infection among MSM. This systematic review followed the Preferred Reporting Items for Systematic Reviews and Meta-Analyses (PRISMA) guidelines [12] and previous approaches in cataloging methods in high-impact literature [13,14]. Our goals were to (1) document reports of potential misclassification of high risk sexual behaviors in observational studies focusing on sexual minority men and HIV transmission, and (2) characterize the use of bias adjustment methods in correcting the proxies for sexual risk behaviors.

We selected 14 widely read journals publishing in areas of HIV infection and diseases, clinical research, and epidemiological methods and searched for potential studies assessing the risk of HIV acquisition using proxy variables to capture condomless anal intercourse and categorizing men based on these proxy measures. These journals included:

- HIV- and STI-focused journals: Acquired Immunodeficiency Syndrome, Journal of Acquired Immunodeficiency Syndrome, Acquired Immunodeficiency Syndrome and Behavior, Sexually Transmitted Disease, Sexually Transmitted Infections,
- Epidemiology-focused journals: American Journal of Epidemiology, Epidemiology, International Journal of Epidemiology, European Journal of Epidemiology, and Annals of Epidemiology,
- General medical journals: New England Journal of Medicine, Journal of American Medical Association, Lancet, and Annals of Internal Medicine.

**Figure 1:**
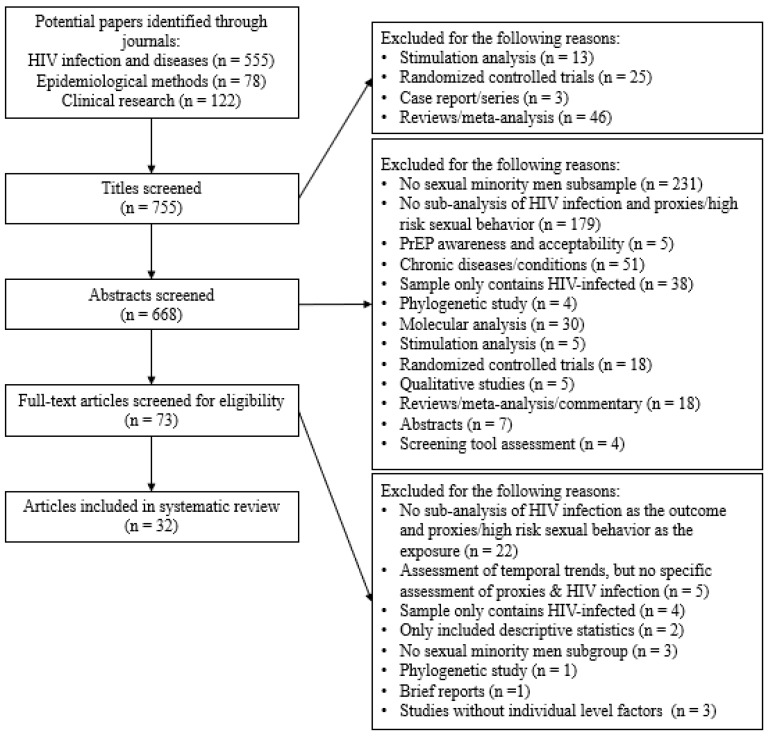
PRISMA Flow Chart of Systematic Search.

We searched through keywords, titles, and abstracts using the following terms (Figure 1): (Men who have sex with men OR MSM OR gay OR homosexual OR bisexual OR same-sex OR high-risk sexual behavior) AND (human immunodeficiency virus OR HIV). To avoid limiting our search results, particularly in epidemiological and medical journals that may have fewer publications among sexual minority men and HIV infection, we broadened the scope of our search and used only the terms men who have sex with men OR HIV. 

### Study Inclusion/Exclusion Criteria

Articles were eligible for inclusion in our study if (1) the publication date was between January 2010 to December 2017; (2) study design was observational; (3) there was a sample/subsample of sexual minority men (i.e., gay, bisexual, or men who have sex with men); (4) high risk sexual behaviors or proxies of such behaviors were evaluated as an exposure of interest; and (5) assessment of HIV infection among sexual minority men as one of the primary outcomes. We excluded studies prior to 2010 to present a description of the current methodology (or lack thereof) in operationalizing high-risk sexual behaviors and potential methods of adjustment when such proxies (e.g., sexual identity) are used. Simulation modeling studies, randomized controlled trials (RCT), and case studies were excluded from our review as these study designs are not subjected to misclassification bias in a similar manner as observation studies: (1) modeling studies draw from previous estimates; therefore, if the estimates itself were not biased then assumptions for models relatively accurate; (2) RCT are less prone to bias as there is greater control exerted by the investigator to accurately measure the primary exposure of interest; and (3) case studies strictly limits itself to pure description; therefore, discussion of inferences does not extend beyond the observations. Also excluded were brief reports, qualitative studies, and studies that did not analyze individual level factors. In addition, we focused on cisgender MSM as they possess the greater burden of HIV infection.

We defined proxies for high risk sexual behaviors as sexual identity, same-sex partner, or same-sex attraction. Same-sex partnerships were classified as ever having sex with a male partner or within the last 12 months. Results were extracted and presented for the following information from each article: (1) how the questionnaire was administered to participants; (2) methods in capturing high risk sexual behaviors such as condomless anal sex; (3) when measurement of such behavior was absent, how MSM was operationalized as the exposure using self-reported proxies for high-risk sexual behaviors such as sexual orientation or gender of sexual partners; (4) acknowledgement of potential misclassification due to inaccurate methodology; and (5) adjustment for purported misclassification of sexual behavior proxy. 

## 3. Results

### 3.1. Assessment of High-Risk Behaviors and MSM Exposure 

The search criteria identified 755 potentially relevant manuscripts. Of these potential papers, 87 (12%) articles were excluded because the title included simulation analysis, RCT, case reports/series, or other systematic reviews/meta-analyses. Among the remaining 668 articles, we further excluded 595 (89%) articles primarily for one of the following reasons: a subsample of sexual minority men was not detected in the study (*n* = 231) or the assessment did not specifically include a sub-analysis evaluating the association of HIV infection with high risk sexual behaviors or proxies (*n* = 179). A total of 73 (10%) articles were retained for full-text examination. Of these, 41 studies (56%) were further removed as they did not have a sub-analysis focusing on sexual risk exposure and HIV infection. Our final sample was 32 (44%) published manuscripts. Studies in this systematic review included diverse MSM populations, including international studies in Asia [15,16,17,18,19,20], Africa [21,22,23], South America [24,25], and Europe [26,27] (41%); Black and Latino persons in the United States [28,29,30,31,32,33,34,35] (25%); and young adults or adolescents [36,37,38,39] (13%). Half of studies (50%) used only community-based surveys [15,16,18,20,21,24,25,28,29,32,35,37,38,40,41,42], 38% (*n* = 12) used population-based surveys [17,19,22,23,26,33,34,36,39,43,44,45], 9% (*n* = 3) used both community and population-based surveys [27,30,31], and one study (3%) used surveillance data to conduct their analysis [46]. All studies were quantitative in nature and assessed the association between HIV infection and various demographic and behavioral characteristics among MSM. 

### 3.2. Self-Reported Proxy of High-Risk Sexual Behavior

Review of each study indicated that self-reported proxy of high-risk sexual behavior used to classify MSM generally fell into one of two categories: sexual identity or partner gender (Table 1). Studies generally used dichotomous (yes/no) questions to ascertain sexual identity and partner gender. These proxies were assessed using self-identified sexual orientation (i.e., heterosexual, gay/homosexual, bisexual, or not sure) or same-sex partners during the previous 12 months or lifetime. In total, 9% (*n* = 3) [34,43,46] used proxies exclusively (i.e., sexual identity or partner gender), 82% (*n* = 26) [15,16,17,18,19,20,21,22,23,25,26,27,28,29,32,33,35,36,37,38,39,40,41,42,44,45] used sexual risk behaviors and did not rely on proxy measures, and 9% (*n* = 3) [24,30,31] used a combination of these measures to evaluate HIV transmission among MSM.

### 3.3. Acknowledgement of Purported Sexual Behavior Misclassification

Among the 32 studies assessing the association of HIV infection with high risk sexual behaviors or proxy variables, 12% (*n* = 4) [28,33,38,39] did not report any potential misclassification and 82% (*n* = 26) [15,16,17,18,19,20,21,22,23,24,25,26,27,29,32,34,35,36,37,40,41,42,43,44,45,46] only discussed misclassification of sexual behavior or MSM classification as a potential limitation. In one such study, clearly stated by Reisner et al. [34], “There was no assessment of recent HIV sexual risk behavior (e.g., unprotected vaginal or anal intercourse) in the NESARC data; therefore, we could not describe or consider recent behavioral risk patterns that may have led to incident HIV infections in our study.” Similar approaches in reporting potential misclassification bias was observed in other articles; however, among these studies, statistical techniques to adjust for misclassification were not employed. 

Two studies (6%) explicitly considered this information bias [30,31]. In the work by Goldstein et al. [31] that examined data from NESARC, the authors noted that MSM had nearly 21 times the odds [95% confidence interval (CI): 6.3, 61.1] of HIV infection compared to non-MSM when classifying higher-risk sexual behavior using self-reported sexual identity. After correcting for potential misclassification of behavior, the authors note this risk estimate was artificially inflated (as men may engage in sex with other men, but not identify as gay/homosexual), and that a more plausible (yet still greatly increased) estimate was approximately a five-fold increase in odds [31]. 

Investigators further assessed racial disparity regarding HIV infection among MSM. In their uncorrected model, MSM were approximately 10 times as likely (95% CI: 5.5, 18) to be infected with HIV compared to non-MSM [30]. Further stratification by race demonstrated that White MSM had greater odds of HIV infection (adjusted prevalence OR = 16; 95% CI: 7.4, 34) compared to non-MSM, whereas Black MSM were 4.5 times as likely (95% CI: 1.4, 12) to acquire HIV compared to non-MSM (a four-fold difference between these racial groups) [30]. After adjusting for misclassification, differences in HIV risk between White and Black MSM in the uncorrected model reduced from a factor of four to two [30]. These results indicated that additional analytic procedures are necessary when using proxies for the exposure (e.g., sexual identity) and outcome (e.g., self-reported HIV infection).

## 4. Discussion

### 4.1. Correcting Risk Estimates

Recent work has summarized techniques for correcting misclassification bias for exposure estimates related to HIV epidemiology [47,48,49]. Goldstein et al. [30,31] demonstrated that using proxy variables may result in inaccurate risk estimation of HIV infection and how risk-adjusting methodologies are an attractive option to arrive at improved assessment of risk when dealing with potentially misclassified risk data that have been previously collected. Furthermore, these techniques demonstrate an intersection of epidemiological methodology and public health practice: research is not occurring in a vacuum but has real-world implications. 

We observed that the majority of reviewed studies did not rely upon proxy exposures, but rather asked specific behavioral questions. Thus, the magnitude of the misclassification may not be as dramatic as first hypothesized. Nevertheless, the potential for misclassification remains as these are observational data. Considering that asking someone intimate details about their sexual acts can lead to prevarication to obscure potentially behaviors, underreporting may lead to biased HIV risk estimation [7,8]. Further, the manner in which questions are asked may elicit varying degrees of misclassification. For example, having an interviewer administer the questions may invoke a social desirability bias, unless trust has been established between the interviewee and interviewer. Therefore, even if the appropriate behavioral questions are asked (with respect to risk for HIV acquisition), there is potential for information bias, which warrants careful consideration by the investigators and use of bias adjustment techniques.

### 4.2. Implications of Biased HIV Risk Data

Consider that an urban health department had data on the HIV epidemic solely from a national survey that reported sexual identity as a proxy for high-risk sexual behavior. The analysis from this survey may demonstrate a strong association between identifying as gay or bisexual and reported HIV infection. Thus, the health department targets its limited funding and resources for HIV testing and prevention campaigns into the city’s gay neighborhood, where the majority of self-identifying lesbian, gay, bisexual, and transgender people live or socialize [50]. While this will undoubtedly have impact, it will also miss populations with substantial risk for infection. The corrected estimates speculate at what might be had the survey included behavioral data, but the analytic methods outlined above cannot elucidate new characteristics. 

Now consider that a health department has data from a survey that has included assessment of specific sexual behaviors in addition to nominal risk group information indicated by self-reported sexual orientation. The analysis may suggest that in addition to self-identified gay men as having an increased risk, there are also pockets of racial minority MSM that have a substantial risk of infection. However, as MSM of color face greater stigma and are less likely to disclose same-sex behavior [51], this underreporting may mask the HIV risk disparity. The use of quantitative bias analysis can assess the degree to which this misclassification is affecting the risk estimates [10]. However, bias correction is not always critical to good policy just as the result of one epidemiological study may never be the sole basis for policy decisions. Observational studies are one of many potential considerations for policy implementation and may sometimes be beneficial to engage in bias adjustment as it provides assurance for the investigators of the internal validity of their analyses. 

### 4.3. Towards Improving the Data

We also use this systematic review as a call to those engaging in primary data collection within sexual minority populations to include detailed behavioral assessment. For example, consider survey items from NESARC, one potential source of nationally generalizable data for HIV infection among sexual minorities [43]. MSM behavior can be inferred from two survey items: one collecting information about self-reported sexual orientation, and a second asking about whether survey respondents have had sex with men. Specific sexual acts are not queried; if a participant indicated he had sex with other men, the risk for HIV is highly dependent upon sexual acts (anal as opposed to oral) and positioning (receptive for greatest risk). A prototypical example comes from the Black and African American Men’s Health Study (BAAMHS): in addition to data on reported sexual identity and partner gender, specific sexual behaviors were also obtained, and while these data more likely ascribe an accurate risk profile, they are limited to non-monogamous black men in the Boston area [52]. 

To improve internal and external validity of findings for HIV and STI evaluations, newer studies are needed with the same level of detail on sexual behaviors as BAAMHS, including: (1) frequency of specific sexual behaviors (oral and/or anal intercourse); (2) positioning (receptive and/or insertive); (3) HIV testing and treatment status; (4) engaging in seroadaptive sex practices, including discussion of HIV infection status and changes of behaviors based on this discussion, including condom use; and (5) information about partnerships to assess sexual networks. Assessing stigmatizing behaviors and conditions via surveys further requires careful consideration for how data are collected in the field. In NESARC, the survey was administered in participants’ homes with research personnel and perhaps even household members present. This is less than ideal, since ensuring some level of privacy for respondents can mitigate underreporting and the social desirability bias leading to differential misclassification of sexual orientation and behaviors. 

Our assessment of recent studies indicates questions pertaining to sexual behavior are more common than proxy exposures, encouraging for HIV risk estimation among sexual minority men. However, as discussed there is still the opportunity for risk estimation to be improved by considering underreporting of risk data. Missing or messy data may potentially fail to provide adequate understanding of the relationship between sexuality and HIV risk. Thus, ascertaining more nuanced information on sexual behaviors and conducting appropriate analyses are needed to make more informed estimation. Yet we also acknowledge the importance of proxy variables such as sexual orientation. These may be appropriate for assessing certain socio-behavioral constructs such as perceived discrimination, stigma, and HIV screening attitude among self-identified gay/bisexual men. 

Our systematic review is subjected to some limitations. It is possible that our search methodology may have missed articles that are pertinent to our aims in describing methods of capturing high risk sexual behaviors. However, the journals that we have selected are high impact journals, thus our results highlight current efforts in the field of HIV risk research and bias correction methods to accurately measure high risk sexual behavior. Secondly, we did not assess misclassification of HIV infection in our review. We understand that measurement error and social desirability bias have the same potential of impacting HIV infection. Previous studies have explored potential solutions for such biases [27,48]. Finally, we are limited to reporting only adjustment techniques that have been presented in the articles. It is possible that investigators are engaging in these analytic methods. If so, we may be underreporting the use of bias analysis techniques. However, absence of misclassification correction techniques should also not be viewed as biased HIV risk estimates.

## 5. Conclusions

While the call for better exposure assessment may not be new [6,11], recent evidence of the magnitude of its bias on HIV transmission risk indicates this is a public health concern that remains. Given the predetermined limitations of how survey items may be collected, analytic methodologies can provide an approach to correct misclassification bias around sexuality and sexual behaviors. However, these techniques can only do so much in the presence of imperfect data. Rather than devoting substantial time and resources to methods to improving data analysis, we should improve the quality of data collected, through more accurate assessment of specific sexual behaviors. In short, it is high-risk behaviors (i.e., receptive anal intercourse) which confer the greatest risk; therefore, questions surrounding sexual behavior (i.e., sexual act and positioning) are of primary importance in surveys that include sexual minority populations. 

## Figures and Tables

**Table 1:** Selected examples of HIV research among men who have sex with men (MSM) and adjustment methods for purported misclassification of sexual behaviors.

Reference	Data Source	Study Description	Population-Based vs. Community-Based Survey	Survey Administration	Proxy Variables		Behavioral Variables		Treatment of Misclassification
					Sexual Identify	Partner Gender	Condomless Anal Intercourse	Position	
Cai et al., 2012 [16]	Cross-sectional survey administered in Shenzhen, China, 2008	Cross-sectional analysis assessing factors associated with HIV prevalence among male sex workers	Community-based Survey	Self-administered questionnaire			X	X	Acknowledgement, but no adjustment
Ko et al., 2011 [15]	Community-based cross-sectional survey, Taiwan, 2004–2008	Cross-sectional analysis of factors associated with HIV/STI	Community-based Survey	Self-administered questionnaire			X		Acknowledgement, but no adjustment
Joseph et al., 2011 [32]	Brothers y Hermanos Study, 2005–2006	Cross-sectional analysis of risk for unrecognized HIV infection among Black and Latino MSM	Community-based Survey	Self-administered computer-based questionnaire			X	X	Acknowledgement, but no adjustment
Oster et al., 2011 [33]	National HIV Behavioral Surveillance Survey (NHBS), 2008	Cross-sectional CI analysis assessing disparities in HIV infection between racial groups	Population-based Survey	Interviewer administered computer-based questionnaire			X		None
Reisner et al., 2011 [34]	National Epidemiologic Survey on Alcohol and Related Conditions (NESARC), 2004–2005	Cross-sectional analysis assessing the association between HIV infection and early life stressors among MSM using a national survey	Population-based Survey	Interviewer administered computer-based questionnaire		X			Acknowledgement, but no adjustment
Walker et al., 2011 [27]	Gay men sexual health survey (GMSHS), British National Survey of Sexual Attitudes and Lifestyles (NATSAL), genitourinary medicine (GUM) clinic	Cross-sectional analysis estimating undiagnosed HIV infection among MSM	Population- and Community-based Survey	Self-administered questionnaire; self-administered computer-based questionnaire; disease reporting system			X		Acknowledgement, but no adjustment
Zhong et al., 2011 [19]	Cross-sectional survey, Guangzhou, China, 2008	Cross-sectional analysis assessing prevalence of HIV among MSM	Population-based Survey	Interviewer administered questionnaire			X	X	Acknowledgement, but no adjustment
Sweet et al., 2012 [43]	National Epidemiologic Survey on Alcohol and Related Conditions (NESARC), 2004–2005	Cross-sectional analysis assessing the association between HIV risk and CSA	Population-based Survey	Interviewer administered computer-based questionnaire	X	X			Acknowledgement, but no adjustment
Wang et al., 2012 [18]	Community based survey, Harbin, China, 2006–2010	Cross-section analysis of HIV prevalence/syphilis and the context of lower rates of condomless anal sex	Community-based Survey	Interviewer administered questionnaire			X		Acknowledgement, but no adjustment
Balaji et al., 2013 [36]	National HIV Behavioral Surveillance Survey (NHBS), 2008	Cross-sectional analysis assessing factors associated with HIV incidence and prevalence	Population-based Survey	Interviewer administered computer-based questionnaire			X	X	Acknowledgement, but no adjustment
Konda et al., 2013 [24]	National Institute of Mental Health (NIMH) Collaborative HIV/STD Prevention Trial Group, 2002–2007	Longitudinal analysis of factors associated with HIV/STI	Community-based survey	Interviewer administered questionnaire		X	X		Acknowledgement, but no adjustment
Sanders et al., 2013 [40]	Prospective cohort study assessing sexual behaviors and HIV/STI acquisition	Longitudinal analysis assessing factors for HIV infection	Community-based Survey	Interviewer administered questionnaire			X	X	Acknowledgement, but no adjustment
Tafuma et al., 2014 [22]	Ministry of Health and Family Health International 360 Survey, Botswana, 2012	Cross-sectional survey analysis assessing factors associated with HIV/STI	Population-based Survey	Interviewer administered questionnaire			X	X	Acknowledgement, but no adjustment
van den Boom et al., 2014 [26]	Amsterdam Cohort Study (ACS), 2007–2011	Cross-sectional survey analysis assessing factors associated with HIV	Population-based Survey	Self-administered questionnaire			X	X	Acknowledgement, but no adjustment
Castillo et al., 2015 [25]	Longitudinal study assessing sexual behaviors and HIV/STI acquisition at baseline, 9- and 18-month follow-up	Discrete time proportional hazard models assessing factors associated with incidence of HIV/STI	Community-based Survey	Interviewer administered questionnaire			X	X	Acknowledgement, but no adjustment
Crosby et al., 2015 [41]	Convenience sample recruited from National Institute of Health—RCT for safer sex intervention, 2012–2014	Cross-sectional analysis assessing differences between HIV-positive and negative MSM in regard to condom use	Community-based Survey	Self-administered questionnaire			X	X	Acknowledgement, but no adjustment
Dailey Garnes et al., 2015 [46]	Sexually Transmitted Disease Management Information System (STD-MIS)	Cross-sectional analysis assessing odds of identifying new HIV infection among social contacts using surveillance data	Neither	Disease reporting system		X			Acknowledgement, but no adjustment
Halkitis et al., 2015 [38]	Prospective cohort study assessing factors associated with HIV infection	Survival analysis assessing factors associated with HIV incidence among young MSM (18–19 years)	Community-based Survey	Interviewer administered questionnaire			X	X	None
Qian et al., 2015 [20]	Cross-sectional survey, Beijing, China, 2010–2011	Cross-sectional analysis assessing risk factors associated with HIV prevalence	Community-based Survey	Self-administered questionnaire				X	Acknowledgement, but no adjustment
Solomon et al., 2015 [17]	Multicenter cross-sectional survey, 12 cities	Cross-sectional analysis assessing prevalence, incidence and factors associated with HIV infection	Population-based Survey	Interviewer administered computer-based questionnaire			X	X	Acknowledgement, but no adjustment
Sullivan et al., 2015 [35]	Prospective cohort study, Atlanta, GA, 2010–2014	Survival analysis assessing racial disparities in HIV infection	Community-based Survey	Self-administered computer-based questionnaire			X	X	Acknowledgement, but no adjustment
Beymer et al., 2016 [28]	Longitudinal study assessing HIV risk factors using a community-based survey, Los Angeles, CA, 2009–2014	Survival analysis assessing factors associated with HIV infection	Community-based Survey	Interviewer administered questionnaire			X	X	None
Eaton et al., 2016 [29]	Cross-sectional survey of Black MSM	Cross-sectional analysis assessing factors associated with testing HIV positive	Community-based Survey	Self-administered computer-based questionnaire			X		Acknowledgement, but no adjustment
Garofalo et al., 2016 [37]	Crew 45—Longitudinal study assessing HIV risk in Chicago for 2 years	Survival analysis assessing factors associated with HIV incidence among young MSM (16–20 years)	Community-based Survey	Self-administered computer-based questionnaire			X		Acknowledgement, but no adjustment
Khosropour et al., 2016 [44]	Data from STD clinic in Seattle & King County, 2001–2013	Retrospective case-control study assessing factors associated with seroconversion and difference in sexual behavior after seroconversion	Population-based Survey	Disease reporting system			X		Acknowledgement, but no adjustment
Davey et al., 2017 [42]	Retrospective study of HIV testing data from LA LGBT Center, 2011–2015	Cross-sectional analysis of MSM assessing factors associated with acute HIV infection	Community-based Survey	Interviewer administered questionnaire			X	X	Acknowledgement, but no adjustment
German et al., 2017 [45]	National HIV Behavioral Surveillance Survey (NHBS), 2008	Cross-sectional analysis of factors associated with HIV transmission risk	Population-based Survey	Interviewer administered computer-based questionnaire			X	X	Acknowledgement, but no adjustment
Goldstein et al., 2015 [31]	National Epidemiologic Survey on Alcohol and Related Conditions (NESARC-2) & Black and African American Men’s Health Study (BAAMHS)	Bayesian analysis for the odds of self-reported HIV infection when adjusted for misclassification of same-sex behavior	Population- and Community-based Survey	Interviewer administered computer-based questionnaire; self-administered computer-based questionnaire	X	X	X	X	Acknowledgement and Bayesian adjustment
Goldstein et al., 2017 [30]	National Epidemiologic Survey on Alcohol and Related Conditions (NESARC-2) & Black and African American Men’s Health Study (BAAMHS)	Bayesian analysis to adjust for residual confounding and correct misclassification of MSM status to help explain racial disparity in HIV infection	Population- and Community-based Survey	Interviewer administered computer-based questionnaire; self-administered computer-based questionnaire	X	X	X	X	Acknowledgement and Bayesian adjustment
Kunzweiler et al., 2017 [21]	The Anza Mapema Study, 2015–2016	Cross-sectional analysis of risk reduction behaviors associated with HIV prevalence among HIV-positive and out of care (vs. HIV negative) and newly diagnosed HIV positive and out of care	Community-based Survey	Self-administered computer-based questionnaire			X		Acknowledgement, but no adjustment
Marano et al., 2017 [39]	NHM&E—National HIV Prevention Program Monitoring and Evaluation system, 2015	Cross-sectional analysis describing linkage to care among new HIV diagnoses and assessing factors associated with HIV incidence	Population-based Survey	Disease reporting system			X		None
Wirtz et al., 2017 [23]	Multi-center cross-sectional survey, 2010–2014	Cross-sectional analysis assessing regional disparities in HIV prevalence and care	Population-based Survey	Interviewer administered questionnaire			X		Acknowledgement, but no adjustment
